# SIRT1 Alleviates Mitochondrial Fission and Necroptosis in Cerebral Ischemia/Reperfusion Injury via SIRT1–RIP1 Signaling Pathway

**DOI:** 10.1002/mco2.70118

**Published:** 2025-02-24

**Authors:** Xuan Wei, Hanjing Guo, Guangshan Huang, Haoyue Luo, Lipeng Gong, Pan Meng, Jiyong Liu, Wenli Zhang, Zhigang Mei

**Affiliations:** ^1^ Key Laboratory of Hunan Province for Integrated Traditional Chinese and Western Medicine on Prevention and Treatment of Cardio‐Cerebral Diseases College of Integrated Traditional Chinese and Western Medicine Hunan University of Chinese Medicine Changsha Hunan China; ^2^ Hunan Provincial Key Laboratory of Traditional Chinese Medicine Diagnostics Hunan University of Chinese Medicine Changsha Hunan China; ^3^ School of Pharmacy Hunan University of Chinese Medicine Changsha Hunan China; ^4^ Third‐Grade Pharmacological Laboratory on Chinese Medicine Approved by State Administration of Traditional Chinese Medicine College of Medicine and Health Sciences China Three Gorges University Yichang Hubei China

**Keywords:** cerebral ischemia/reperfusion injury, DRP1, mitochondrial fission, necroptosis, RIP1, SIRT1

## Abstract

Programmed cell death, including necroptosis, plays a critical role in the pathogenesis of cerebral ischemia/reperfusion injury (CIRI). Silent information regulator 1 (SIRT1) has been identified as a potential therapeutic target for CIRI, yet its precise role in regulating necroptosis remains controversial. Furthermore, the potential interaction between SIRT1 and receptor‐interacting protein kinase 1 (RIP1) in this context is not fully understood. *Sanpian* Decoction (SPD), a classical traditional herbal formula, was previously shown to enhance SIRT1 expression in our studies. Our findings demonstrated that, both in vivo and in vitro, CIRI was associated with a decrease in SIRT1 levels and phosphorylated dynamin‐related protein 1 (p‐DRP1) at Ser637, alongside an increase in RIP1 and other necroptosis‐related proteins. Co‐immunoprecipitation and immunofluorescence analyses revealed a weakened interaction between SIRT1 and RIP1. Furthermore, abnormal mitochondrial fission and dysfunction were mediated through the phosphoglycerate mutase 5–DRP1 pathway. Notably, SPD treatment improved neurological outcomes and reversed these pathological changes by enhancing the SIRT1–RIP1 interaction. In conclusion, this study suggests that SIRT1 is a promising therapeutic target for CIRI, capable of inhibiting necroptosis and mitigating mitochondrial fission via the SIRT1–RIP1 pathway. SPD exhibits therapeutic potential by activating SIRT1, thereby attenuating necroptosis and mitochondrial fission during CIRI.

## Graphical abstract

SIRT1, a NAD+‐dependent protein deacetylase, is widely distributed in brain tissue. When cerebral ischemia/reperfusion injury (CIRI) occurs, downregulation of SIRT1 weakens its interaction with RIP1, facilitating necrosomes formation, and thus exacerbates necroptosis. Additionally, the downstream target PGAM5 dephosphorylates DRP1 at Ser637, worsening mitochondrial fission and dysfunction, and further aggravates CIRI. *Sanpian* Decoction (SPD), a Chinese herbal formula, serves as a SIRT1 activator, protects against CIRI via the SIRT1–RIP1 pathway

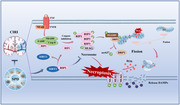

## 1 Introduction

Ischemic stroke, a cerebrovascular event, is one of the leading causes of death and disability globally, accounting for 70–80% of all stroke cases [1, 2]. Currently, reperfusion strategies, including pharmacological thrombolysis and mechanical thrombectomy, are recommended as the standard treatments for acute ischemic stroke [3]. Tissue plasminogen activator (t‐PA) is the only thrombolytic agent approved by the United States Food and Drug Administration for ischemic stroke treatment. However, the use of t‐PA is limited by its narrow therapeutic window and the risk of hemorrhagic transformation. Additionally, although blood flow recanalization is crucial, it can paradoxically exacerbate the neural injury and neurological dysfunction caused by the initial ischemic insult—a phenomenon known as cerebral ischemia/reperfusion injury (CIRI). CIRI has become a critical factor in determining the prognosis and recovery of patients after ischemic stroke. Given the complex pathological mechanisms underlying CIRI, current therapeutic strategies have not achieved the desired effects. Therefore, it is essential to further explore the mechanisms involved in CIRI to develop more effective treatments for ischemic stroke.

Accumulating evidence suggests that mitochondrial dysfunction, particularly the imbalance in mitochondrial dynamics, plays a crucial role in programmed cell death during CIRI [4, 5, 6]. Reduced expression of phosphorylated dynamin‐related protein 1 (p‐DRP1) at Ser637, p‐DRP1(s637) [7], along with lower levels of optic atrophy 1 and mitofusion 1/2 (MFN1/2), and an elevated level of p‐DRP1 at Ser616 [8] can disrupt mitochondrial dynamics, promote fission, increase fragmentation, and ultimately induce mitochondrial dysfunction, exacerbating programmed cell death. Necroptosis is a distinct form of programmed cell death that is independent of caspases, characterized by the morphological features of necrosis and pathways akin to apoptosis [9]. Although necroptosis can be triggered by various upstream signals, such as tumor necrosis factor receptors (TNFR), factor‐related apoptosis (FAS), and pathogen recognition receptors [10], the pathway converges on the activation of receptor‐interacting protein 1/3 (RIP1/3) and phosphorylated mixed lineage kinase domain‐like protein (p‐MLKL). RIP1 is activated when it dissociates from its original complex in response to tumor necrosis factor α (TNFα) signaling. Phosphorylated RIP1 first acts as a kinase, interacting with RIP3 to form necrosomes, which recruit and phosphorylate MLKL. These necrosomes then translocate to the cell membrane, altering osmotic pressure and triggering the release of damage‐associated molecular patterns (DAMPs), which can cause cell swelling, rupture, or death [9]. Phosphoglycerate mutase 5 (PGAM5), a downstream signal in necroptosis, is activated by the RIP1–RIP3–p‐MLKL signal. PGAM5 recruits dynamin‐related protein 1 (DRP1) to the outer mitochondrial membrane, leading to dephosphorylation of p‐DRP1(s637), which initiates mitochondrial fission during cardiac ischemia–reperfusion injury [11]. It has been reported that RIP1–RIP3–p‐MLKL levels are elevated in a mouse model of middle cerebral artery occlusion (MCAO) [12]. However, the role of the PGAM5–DRP1 pathway in linking mitochondrial fission and necroptosis during CIRI remains unexplored.

Silent information regulator 1 (SIRT1), a nicotine adenine dinucleotide+‐dependent enzyme and a member of the sirtuin family, is widely distributed in the brain, with high expression in the hippocampus, cerebellum, cortex, and hypothalamus. Initially described as a nuclear protein, SIRT1 can also shuttle to the cytoplasm during neuronal differentiation and synaptic growth [13]. It has been reported that SIRT1 prevents lysine acetylation of hypoxia‐inducible factor‐1α (HIF‐1α), thereby restoring the balance between glycolysis and oxidative phosphorylation [14]. Additionally, SIRT1 modulates mitogen‐activated protein kinase (MAPK) signaling, alleviating oxidative stress, mitochondrial dysfunction, and cell death by decreasing phosphorylation of p38 and c‐Jun N‐terminal kinase, while increasing phosphorylation of extracellular signal‐regulated kinase (ERK) [15]. Interestingly, promoting SIRT1 expression has been shown to mitigate necroptosis in skeletal muscle compression injury [16]. Moreover, resveratrol, a natural polyphenol and potent SIRT1 activator, has been found to exert protective effects in vibrio vulnificus‐induced sepsis by inhibiting necroptosis [17]. Tristetraprolin (TTP) activation via the adenosine 5'‐monophosphate (AMP) activated protein kinase (AMPK)–SIRT1 pathway has been shown to decrease TNFα levels in Kupffer cells, subsequently reducing RIP1 expression and alleviating necroptosis [18]. However, the role of SIRT1 in regulating necroptosis during CIRI remains controversial. In a rat model of MCAO/reperfusion (MCAO/R) rat model, upregulation of SIRT1 has been shown to be associated with increased necroptosis [19], contradicting the protective role of SIRT1 reported in other disease contexts. This conflicting evidence highlights the need for further investigation into SIRT1's specific role in necroptosis during CIRI. Notably, previous research has identified two RIP1 deacetylation sites regulated by SIRT1. Inhibition of SIRT1 increases acetylation of the death domain in RIP1, thereby influencing RIP1‐mediated cell death [20]. However, whether the interaction between SIRT1 and RIP1 directly contributes to the molecular mechanisms of necroptosis remains unclear and warrants further study.

*Sanpian* decoction (SPD), a classical Chinese herbal formula composed of eight herbs, is widely used in clinical practice to treat acute ischemic stroke and migraine [21, 22]. Our previous studies have demonstrated that SPD effectively reduces CIRI‐induced inflammation and oxidative stress by upregulating SIRT1 expression [23, 24]. High‐performance liquid chromatography and ultra‐high‐performance liquid chromatography were utilized to analyze the chemical constituents of SPD. In combination with network pharmacological approaches, these methods confirmed that SPD exerted therapeutic effects by targeting multiple pathways, including MAPK, HIF‐1α, and TNFα. SPD's role as a potent activator of SIRT1 in mediating antiapoptotic effects has been preliminarily elucidated, functioning through the SIRT1/ERK/HIF‐1α signaling cascade [25]. However, its role in regulating necroptosis remains unclear, and the precise mechanisms by which SPD alleviates necroptosis in neuronal cells and interferes with the interaction between mitochondrial fission and necroptosis have not been fully elucidated.

In this study, we focused on SIRT1, a ubiquitously expressed deacetylase in the brain, which had been shown to improve cognitive function and reduce pathological lesions in vitro, as well as inhibit cell death and improve mitochondrial morphology and function in vivo. Mechanistically, the downregulation of SIRT1 weakens its interaction with RIP1, facilitating necrosome formation and thus exacerbating necroptosis. Furthermore, the downstream target PGAM5 dephosphorylates and activates DRP1 at Ser637, promoting mitochondrial fission and dysfunction. SPD, acting as an activator of SIRT1, reverses these pathological changes via the SIRT1–RIP1 pathway. Therefore, this study aims to elucidate the mechanisms underlying the interaction between SIRT1 and necroptosis and explore the therapeutic potential of traditional Chinese medicine (TCM), specifically SPD, in targeting SIRT1 as a treatment strategy for ischemic stroke.

## 2 Results

### 2.1 SIRT1 Activator Recovered Cerebral Blood Flow and Ameliorated Cognitive Impairment in Vivo

**FIGURE 1:**
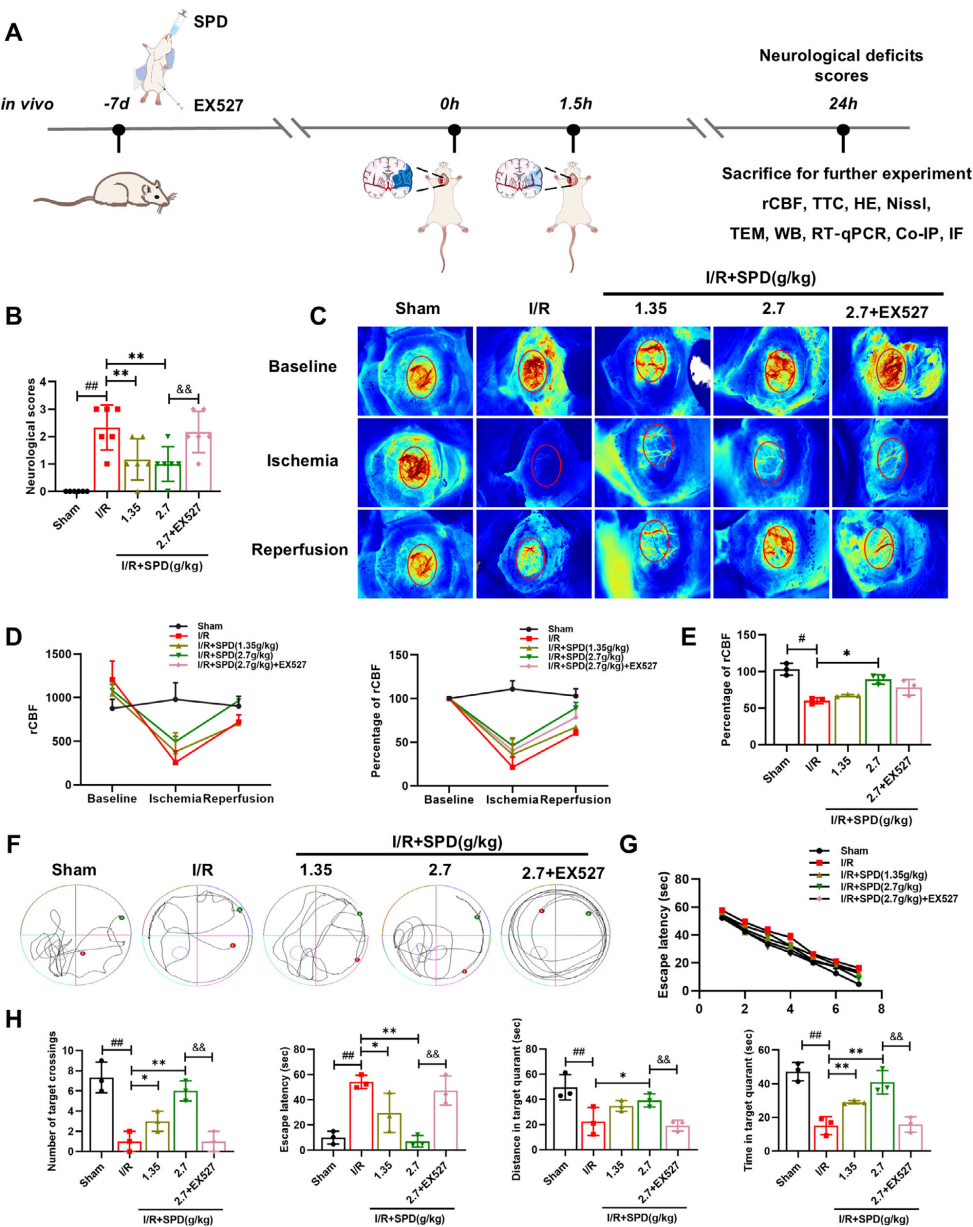
Effects of SIRT1 activator on cerebral blood flow and cognitive impairment in vivo. (A) The experimental protocol in vivo. (B) Neurological scores in rats after 24 h of reperfusion (*n* = 6). (C) Representative laser speckle images of cerebral blood flow at pre‐operation (baseline), occlusion (ischemia), and reperfusion. Red labeling: regional cerebral blood flow (rCBF). (D) The value of rCBF and the percentage of rCBF in the ischemic hemisphere in line graphs. (E) The percentage of rCBF of the ischemic hemisphere after operation (*n* = 3). (F) Representative swimming traces from five groups of rats during probe trials on day 7. (G) Latency to reach the hidden platform during training trials. (H) The data of Morris water maze test (*n* = 3). The results are expressed as the mean ± SEM. ^#^ *p* < 0.05, ^##^ *p* < 0.01; sham vs. ischemia/reperfusion (I/R). ^*^ *p* < 0.05, ^**^ *p* < 0.01; I/R vs. I/R+SPD. ^&&^ *p* < 0.01; I/R+SPD (2.7 g/kg) vs. I/R+SPD (2.7 g/kg) + EX527.

To evaluate the effect of the SIRT1 activator on cerebral blood flow and neurological function, laser speckle analysis and Morris water maze were assessed in vivo (Figure 1A). Pretreatment with SPD at a dose of 2.7 g/kg significantly reduced Longa scores, indicating an improvement in neurological function (Figure 1B). Regional cerebral blood flow (rCBF) was monitored at three key points during the surgical procedure: baseline, ischemia, and reperfusion (Figure 1C). In the sham group, both rCBF values and rCBF percentages remained stable throughout the experiment, with minimal deviation from baseline levels. However, upon the induction of MCAO, rCBF in the ischemic hemisphere significantly decreased. Following the removal of the nylon thread, a notable recovery in rCBF was observed at 24 h of reperfusion (Figure 1D). As shown in Figure 1D,E, postreperfusion rCBF in the SPD (2.7 g/kg) group significantly increased compared with the I/R group. Additionally, the Morris water maze test was performed to assess spatial learning and memory following MCAO/R surgery. Rats in the SPD‐treated groups, particularly those in the SPD (2.7 g/kg) group, showed significant improvements in cognitive function (Figure 1F,G). These improvements included shorter latency to the first platform crossing, increased time spent in the target quadrant, and a higher total number of platform crossings (Figure 1H). However, the beneficial effects of SPD pretreatment were abolished by the SIRT1 inhibitor EX527.

### 2.2 SIRT1 Activator Attenuated Pathological Lesions in the Cerebral Cortex and the CA1 Region of the Hippocampus

**FIGURE 2:**
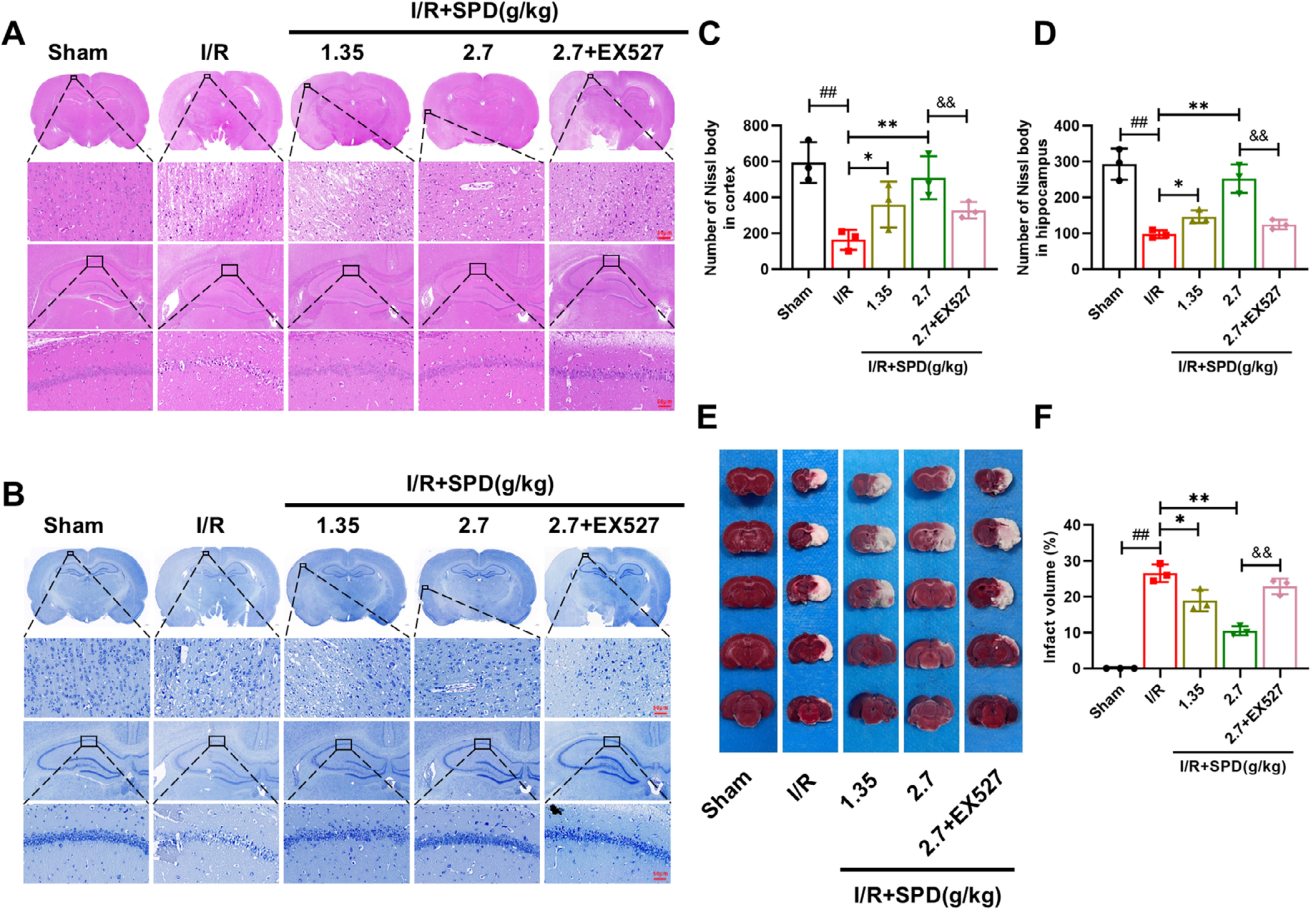
Effects of SIRT1 activator on pathological lesions in vivo. (A) Observation of the morphology in cortex of ischemic penumbra (×400, scale bar: 50 µm) and in the CA1 region of the hippocampus (×400, scale bar: 50 µm) by hematoxylin and eosin (HE) staining. The boxed regions in the upper panels were enlarged into the lower panels. (B) Observation of the morphology in cortex of ischemic penumbra (×400, scale bar: 50 µm) and in the CA1 region of the hippocampus (×400, scale bar: 50 µm) by Nissl staining. The boxed regions in the upper panels were enlarged into the lower panels. (C and D) The number of Nissl bodies. (E) Photographs of 2,3,5‐triphenyltetrazolium chloride (TTC)‐stained brain slices. (F) The percentage of the infarct volume (*n* = 3). The results are expressed as the mean ± SEM. ^##^ *p *< 0.01; sham vs. I/R. ^*^ *p *< 0.05, ^**^ *p *< 0.01; I/R vs. I/R+SPD. ^&&^ *p *< 0.01; I/R+SPD (2.7 g/kg) vs. I/R+SPD (2.7 g/kg) + EX527.

To evaluate morphological changes in MCAO/R rats, hematoxylin and eosin (HE) staining, Nissl staining, and 2,3,5‐triphenyltetrazolium chloride (TTC) staining were performed on the cerebral cortex of ischemic penumbra and the CA1 region of the hippocampus. As depicted in Figure 2A (HE staining) and Figure 2B (Nissl staining), the I/R group exhibited significant neuronal loss and tissue damage in both the cortex and CA1 region. The results of HE staining revealed a reduced number of cortical neurons with deeply stained, pyknotic nuclei, indicative of cell death. Additionally, disorganized cellular architecture and vacuolation were observed in the CA1 region. In contrast, SPD pretreatment significantly mitigated these pathological lesions. Notably, at a dose of 2.7 g/kg, SPD markedly improved neuronal recovery compared with the MCAO/R group, as demonstrated by the increased number of Nissl bodies in both the cortex and CA1 region (Figure 2C,D). Consistent with these findings, Figure 2E,F showed that cerebral infarct volumes were reduced in SPD‐treated rats, with the higher dose (2.7 g/kg) exhibiting more pronounced effects. These results suggest that SPD, a potent SIRT1 activator, exerts significant anti‐CIRI effects, warranting further investigation as a potential therapeutic agent.

### 2.3 The Upregulation of SIRT1 Protected Against CIRI by Hindering the Necroptosis in Vivo

**FIGURE 3:**
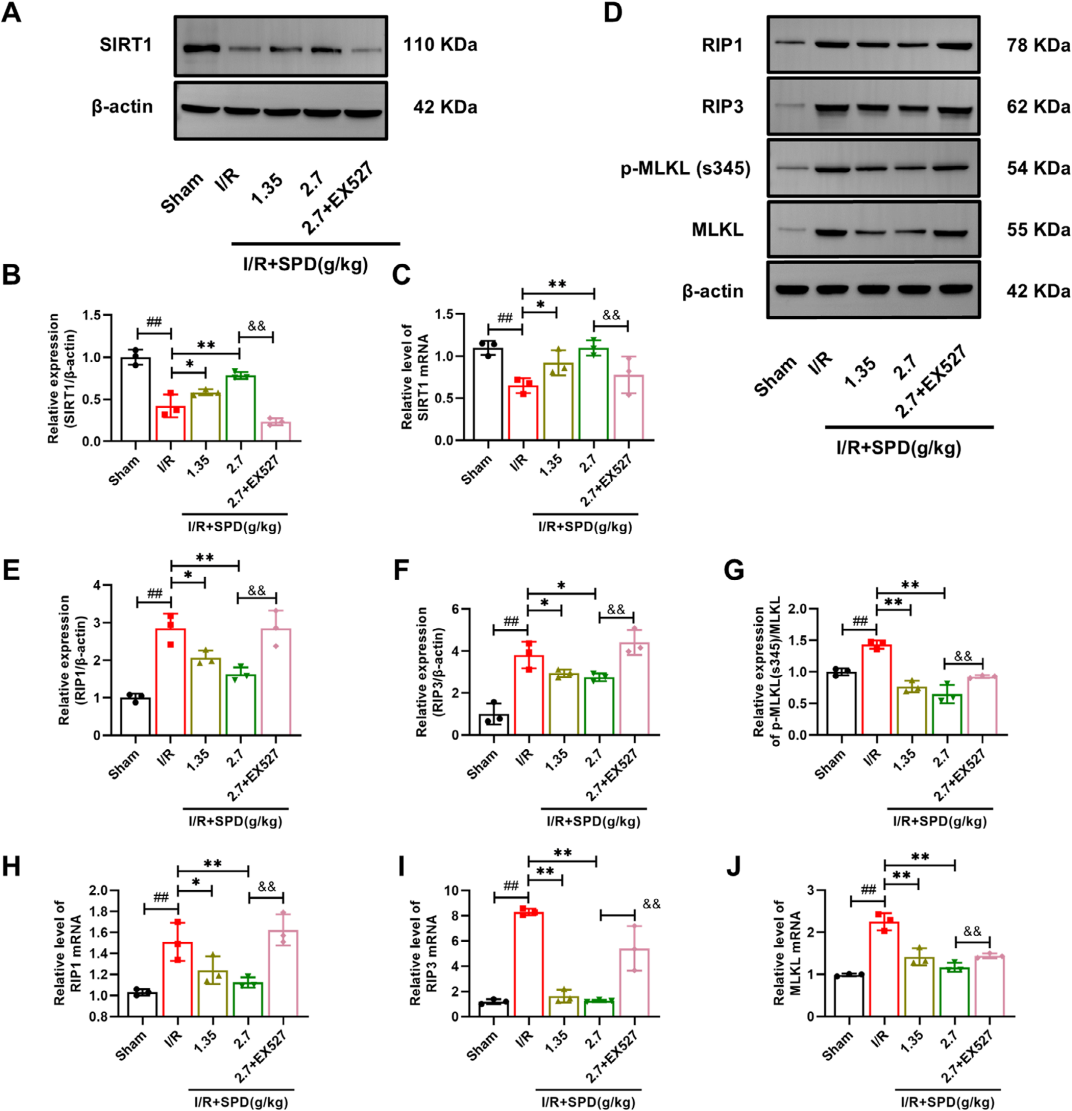
Effects of SIRT1 on the necroptosis in vivo. (A and B) Representative blots and quantitative analysis of SIRT1. (C) The mRNA level of SIRT1. (D–G) Representative blots and quantitative analysis of RIP1, RIP3, p‐MLKL(s345)/MLKL. (H–J) The mRNA levels of RIP1, RIP3, and MLKL (*n* = 3). The results are expressed as the mean ± SEM. ^##^ *p *< 0.01; sham vs. I/R. ^*^ *p *< 0.05, ^**^ *p *< 0.01; I/R vs. I/R+SPD. ^&&^ *p *< 0.01; I/R+SPD (2.7 g/kg) vs. I/R+SPD (2.7 g/kg) + EX527.

To explore the underlying mechanism of SIRT1 to alleviate CIRI, Western bolt and real‐time quantitative PCR (RT‐qPCR) were undertaken. As illustrated in Figure 3A–C, both the protein and messenger ribonucleic acid (mRNA) levels of SIRT1 were significantly decreased in the I/R group. Meanwhile, the levels of RIP1, RIP3, and p‐MLKL/MLKL were upregulated in the I/R group, indicating a marked induction of necroptosis following CIRI (Figure 3D–J). To investigate whether SIRT1 influences SPD's effectiveness in alleviating necroptosis, rats were pretreated with SPD or the SIRT1 inhibitor EX527, and the expression of necroptosis‐related proteins was analyzed. SPD pretreatment significantly decreased the levels of RIP1, RIP3, and p‐MLKL/MLKL, as well as their mRNA levels, in the I/R group. However, EX527 prevented SPD‐induced increases in SIRT1 expression and blocked the reduction of RIP1, RIP3, and p‐MLKL/MLKL levels. The inhibition of SIRT1 by EX527 negated the neuroprotective effects of SPD, indicating that SPD's efficacy in reducing necroptosis and improving neurological deficiency is, to a significant extent, dependent on SIRT1 activation.

### 2.4 SPD Alleviated Oxygen‐Glucose Deprivation/Recovery‐Induced Damage in Vitro

**FIGURE 4:**
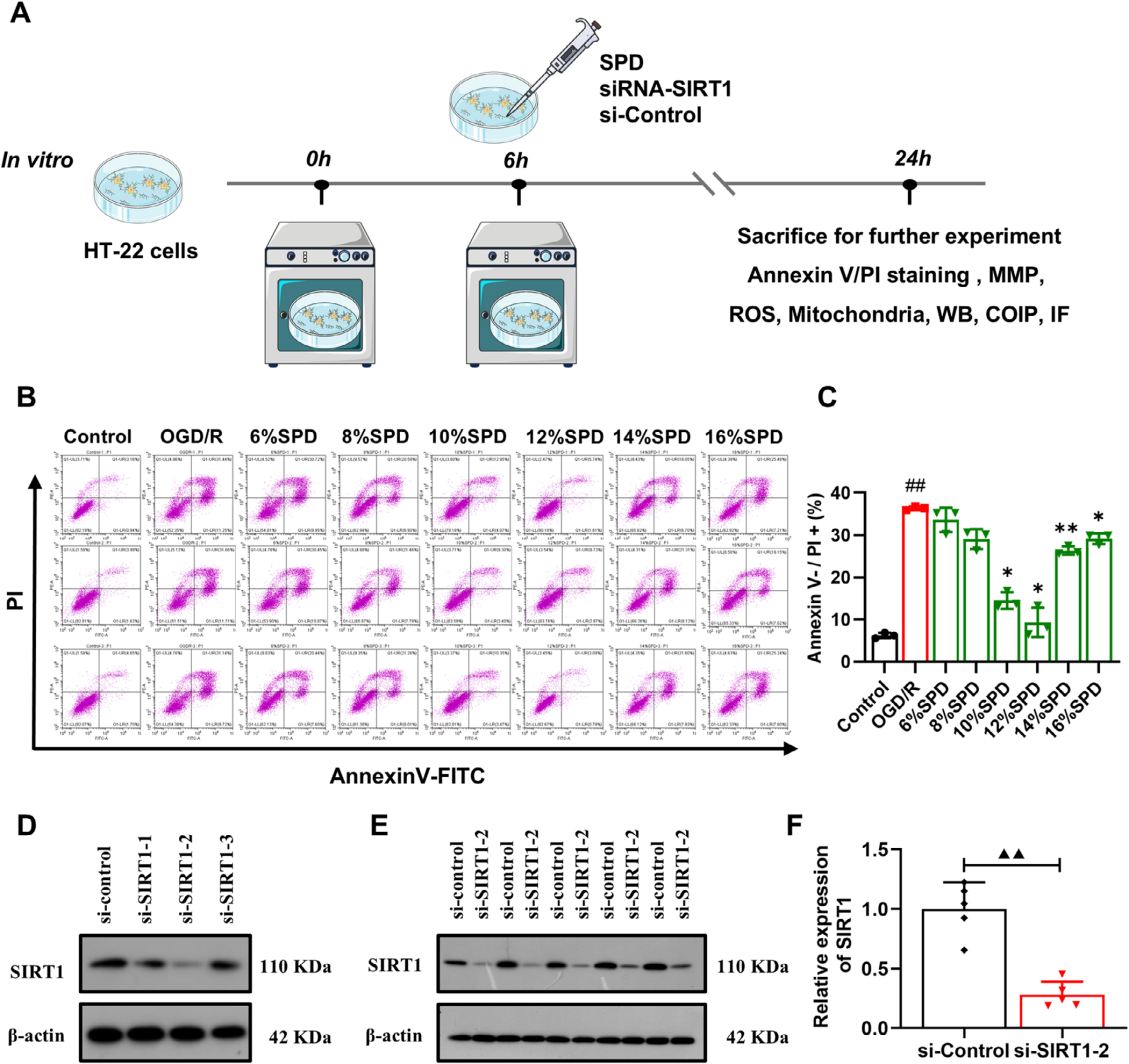
Optimization of drug‐containing serum dose and si‐SIRT1 silencing efficiency. (A) The experimental protocol in vitro. (B and C) Annexin V and PI staining under different doses of SPD. PI+ cells represented necrosis (*n* = 3). (D) Western blot validation of si‐SIRT1 silencing efficiency. (E and F) Representative blots and quantitative analysis of si‐SIRT1‐2 (*n* = 5). The results are expressed as the mean ± SEM. ^##^ *p *< 0.01; control vs. oxygen‐glucose deprivation/recovery (OGD/R). ^*^ *p *< 0.05, ^**^ *p *< 0.01; OGD/R vs. OGD/R+SPD. ^▲▲^ *p *< 0.01; si‐Control vs. si‐SIRT1‐2.

The effect of SPD on oxygen‐glucose deprivation/recovery (OGD/R)‐induced injury in HT‐22 cells was evaluated using Annexin V/PI double staining (Figure 4A). HT‐22 cells subjected to OGD/R exhibited a significant increase in both necroptosis (Figure 4B,C) and apoptosis (Figure S1). However, as shown in Figure 4B, pretreatment with SPD at various concentrations significantly alleviated cellular injury, indicating its protective effects against OGD/R‐induced damage. Based on a previous study, which identified the most pronounced improvement in cell viability with SPD at 5.4 g/kg [25], we tested a range of SPD concentrations (5.4 g/kg, 6, 8, 10, 12, 14, 16%) on OGD/R‐treated HT‐22 cells. For subsequent experiments, the optimal concentration of SPD was determined to be 12% (5.4 g/kg), as this dose produced the most significant improvement in cell viability by reducing both necroptosis and apoptosis.

As illustrated in Figure 4D–F, Western blot analysis confirmed the silencing efficiency of SIRT1 using small interfering RNA (siRNAs). The si‐SIRT1‐1, si‐SIRT1‐2, and si‐SIRT1‐3 groups all demonstrated a reduction in SIRT1 protein levels compared with the si‐Control group. And qPCR detection was utilized to detect si‐SIRT1 interference efficiency (Figure S2A,B and Tables S1 and S2). Among these, si‐SIRT1‐2 showed the highest silencing efficiency, making it the optimal choice for subsequent experiments.

### 2.5 SIRT1–RIP1 Pathway Mitigated the Necroptosis in Vitro

**FIGURE 5:**
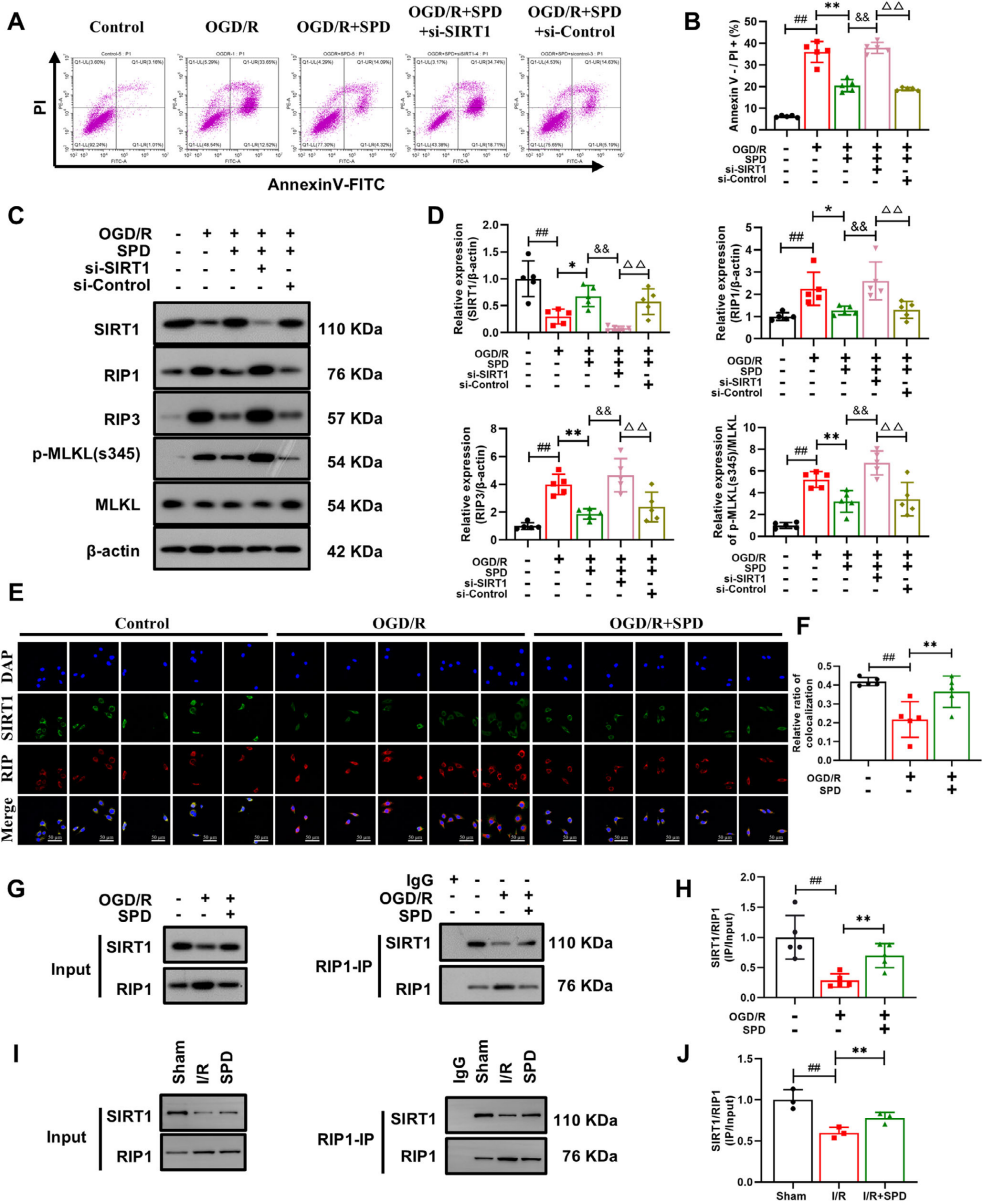
Effects of SIRT1 upregulation on the SIRT1–RIP1 pathway in vitro. (A and B) Representative images and analysis of Annexin V and PI staining. PI+ cells represented necrosis. (C and D) Representative blots and quantitative analysis of SIRT1, RIP1, RIP3, p‐MLKL(s345)/MLKL. (E and F) Immunofluorescence of SIRT1 colocalized with RIP1 (scale bar: 50 µm). (G and H) Analysis of the changes in the binding ability of SIRT1 and RIP1 by co‐immunoprecipitation in HT‐22 cells (*n* = 5). The results are expressed as the mean ± SEM. ^##^ *p *< 0.01; control vs. OGD/R. ^*^ *p *< 0.05, ^**^ *p *< 0.01; OGD/R vs. OGD/R+SPD. ^&&^ *p *< 0.01; OGD/R+SPD vs. OGD/R+SPD+si‐SIRT1. ^△△^ *p *< 0.01; OGD/R+SPD+si‐SIRT1 vs. OGD/R+SPD+si‐Control. (I and J) Analysis of the changes in the binding ability of SIRT1 and RIP1 by co‐immunoprecipitation in rats (*n* = 3). The results are expressed as the mean ± SEM. ^##^ *p *< 0.01; sham vs. I/R. ^**^ *p *< 0.01; I/R vs. I/R+SPD.

As depicted in Figure 5A,B, the activation of SIRT1 significantly alleviated OGD/R‐induced necroptosis in a SIRT1‐dependent manner in HT‐22 cells. However, when *SIRT1* was silenced using siRNA, the rate of necroptosis returned to levels comparable to OGD/R alone. Consistent with previous findings, SPD was effective in reducing apoptosis via SIRT1 activation (Figure S3) [25]. Western blot analysis revealed that SIRT1 protein levels were upregulated, while the levels of RIP1, RIP3, and p‐MLKL/MLKL were significantly decreased. However, in cells pretreated with SIRT1 silencing, these effects were reversed, opposing the impact seen with SPD treatment alone or si‐Control (Figure 5C,D).

Previous evidence has shown that acetyltransferase/deacetylase enzymes can regulate RIP1 through (de)acetylation, and the interaction between SIRT1 and RIP1 plays a key role in controlling cell death [20]. Then, immunofluorescence (IF) and co‐immunoprecipitation (Co‐IP) techniques were employed to further explore the involvement of SIRT1 in the regulation of necroptosis. As illustrated in Figure 5E,F, IF labeling demonstrated colocalization of SIRT1 and RIP1 in normal HT‐22 cells. The relative colocalization of these two proteins significantly decreased in OGD/R cells but was restored with SPD pretreatment. The Co‐IP results further confirmed a significant increase in the interaction between SIRT1 and RIP1 in vitro (Figure 5G,H) and in vivo (Figure 5I,J) following SPD administration, suggesting that this interaction exerts a crucial and physiologically relevant role in the neuroprotective effects mediated by SIRT1 activation. Considering that total SIRT1 protein levels were similarly altered, the results presented in Figure 5H,J indicate that the increased complexation of SIRT1 and RIP1 in SPD‐treated I/R injury models is likely due to an enhanced affinity between the two proteins. In conclusion, SIRT1, through its interaction with RIP1, acts as a suppressor of CIRI‐induced necroptosis.

### 2.6 SIRT1 Upregulation Restored Mitochondrial Function and Morphology

**FIGURE 6:**
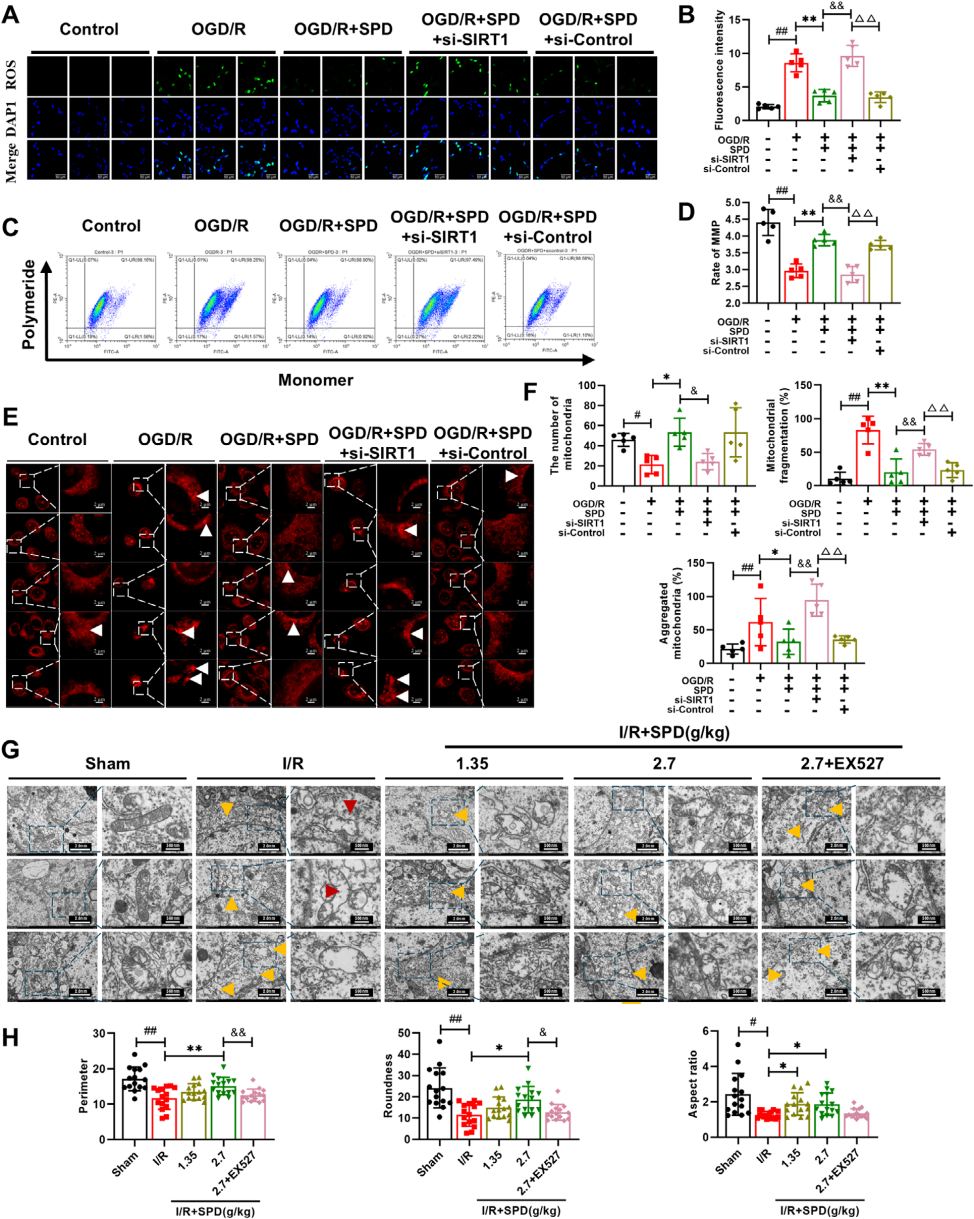
Effects of SIRT1 on mitochondrial function and mitochondrial fission. (A and B) Fluorescence images and levels of reactive oxygen species (ROS) (Scale bar: 50 µm). (C and D) Representative images and levels of mitochondrial membrane potential (MMP). (E) Fluorescence images of mitochondrial in HT‐22 cells. The boxed regions in the left panels were enlarged into the right panels. White arrow: aggregated mitochondrial. (F) Quantitative analysis of mitochondrial number, mitochondrial fragmentation rate, and aggregated mitochondrial rate (*n* = 5). (G) Morphology of the mitochondria under transmission electron microscopy (TEM) in vivo (original magnification: ×7000; scale bar: 2.0 µm × 20,000; scale bar: 500 nm). 15 fields of view were selected separately for each group. The boxed regions in the left panels were enlarged into the right panels. Yellow arrow: damaged mitochondria; red arrow: mitochondrial fission. (H) Quantitative analysis of mitochondrial perimeter, roundness, and aspect ratio (*n* = 15). The results are expressed as the mean ± SEM. ^#^ *p *< 0.05, ^##^ *p *< 0.01; control vs. OGD/R. ^*^ *p *< 0.05, ^**^ *p *< 0.01; OGD/R vs. OGD/R+SPD. ^&^ *p *< 0.05, ^&&^ *p *< 0.01; OGD/R+SPD vs. OGD/R+SPD+si‐SIRT1. ^△^ *p* < 0.05, ^△△^ *p *< 0.01; OGD/R+SPD+si‐SIRT1 vs. OGD/R+SPD+si‐Control.

As mitochondria are central to cellular energy metabolism, we assessed mitochondrial function and morphology using JC‐1 flow cytometry and confocal microscopy to visualize reactive oxygen species (ROS) and mitochondrial structure (Figure 6). The results demonstrated a significant increase in ROS levels and a decrease in mitochondrial membrane potential (MMP) in HT‐22 cells exposed to OGD/R. In contrast, SIRT1 upregulation significantly restored MMP levels and reduced ROS accumulation in OGD/R‐treated cells. However, the silencing of the *SIRT1* gene in these cells prevented any improvement in mitochondrial dysfunction (Figure 6A–D). Additionally, we examined whether SIRT1 could inhibit OGD/R‐induced mitochondrial fragmentation in HT‐22 cells by visualizing mitochondrial morphology using the MitoTracker Red probe. As presented in Figure 6E,F, mitochondria in normal HT‐22 cells appeared as elongated tubules forming highly interconnected networks. Upon OGD/R stimulation, confocal images revealed that mitochondria became spherical and fragmented. Notably, SIRT1 upregulation attenuated these OGD/R‐induced morphological changes, as evidenced by an increase in mitochondrial number and a reduction in mitochondrial fragmentation and aggregation. Transmission electron microscopy (TEM) was undertaken to observe mitochondrial morphology in vivo. As indicated by the arrows in Figure 6G, MCAO/R caused significant mitochondrial damage, including vacuolar degeneration, swelling, cristae loss, and mitochondrial fission. SPD treatment (2.7 g/kg) significantly attenuated these morphological injuries, as reflected by an increase in mitochondrial perimeter, roundness, and aspect ratio (Figure 6H). However, *SIRT1* silencing reversed these protective effects, negating the benefits observed with SPD treatment.

### 2.7 SIRT1 Silencing Abolished the Effects of SPD on the PGAM5–DRP1 Pathway in Mitochondrial Fission in Vitro and in Vivo

**FIGURE 7:**
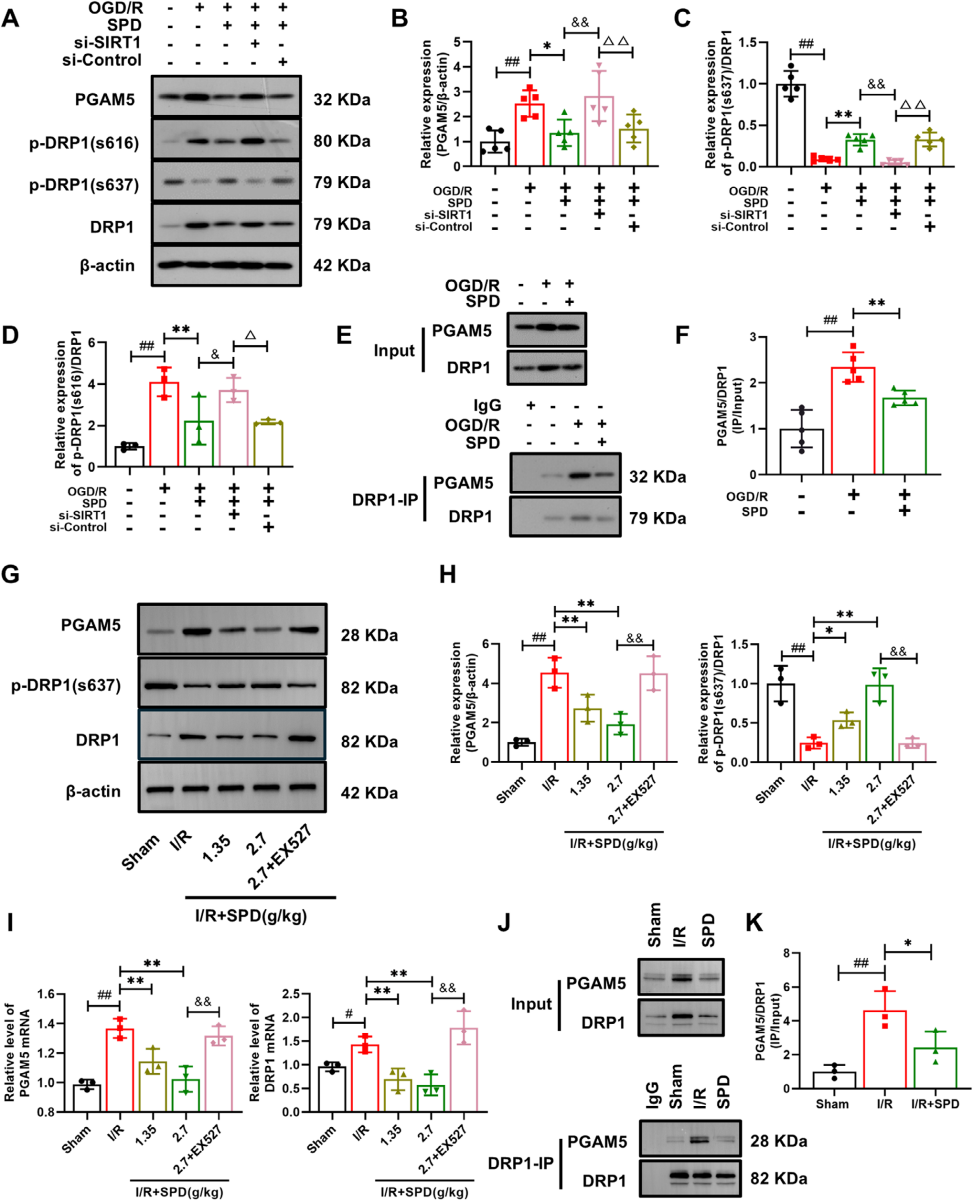
Effects of SIRT1 and PGAM5–DRP1 on mitochondrial fission. (A–D) Representative blots and quantitative analysis of PGAM5, p‐DRP1(s637)/DRP1 in vitro (*n* = 5), and p‐DRP1(s616)/DRP1 (*n* = 3). (E and F) Analysis of the changes in the binding ability of PGAM5 and DRP1 by co‐immunoprecipitation in HT‐22 cells (*n* = 5). ^##^ *p *< 0.01; control vs. OGD/R. ^*^ *p *< 0.05, ^**^ *p *< 0.01; OGD/R vs. OGD/R+SPD. ^&^ *p *< 0.05, ^&&^ *p *< 0.01; OGD/R+SPD vs. OGD/R+SPD+si‐SIRT1. ^△^ *p* < 0.05, ^△△^ *p *< 0.01; OGD/R+SPD+si‐SIRT1 vs. OGD/R+SPD+si‐Control. (G and H) Representative blots and quantitative analysis of PGAM5, p‐DRP1(s637)/DRP1 in vivo. (I) The mRNA levels of PGAM5 and DRP1. (J and K) Analysis of the changes in the binding ability of PGAM5 and DRP1 by co‐immunoprecipitation in rats (*n* = 3). The results are expressed as the mean ± SEM. ^#^ *p *< 0.05, ^##^ *p *< 0.01; sham vs. I/R. ^*^ *p *< 0.05, ^**^ *p *< 0.01; I/R vs. I/R+SPD. ^&^ *p *< 0.05, ^&&^ *p *< 0.01; I/R+SPD (2.7 g/kg) vs. I/R+SPD (2.7 g/kg) + EX527.

The association between PGAM5 and the classical mitochondrial fission‐related protein DRP1 has been investigated previously, but its role in CIRI remains unclear. To further elucidate the association between PGAM5 and DRP1 and to explore the underlying mechanisms, we conducted Western blot and Co‐IP assays to detect PGAM5 and DRP1 in vitro. As shown in Figure 7A–C, OGD/R‐induced cells exhibited increased expression of PGAM5 and decreased p‐DRP1(s637)/DRP1 levels. Co‐IP analysis indicated that DRP1 interacted with PGAM5, and this interaction was enhanced following OGD/R injury (Figure 7E,F). To assess the impact of SIRT1 on mitochondrial fragmentation and fission regulation, we repeated the experiments with SPD administration in vitro and in vivo. SPD administration inhibited the interaction between PGAM5 and DRP1 (Figure 7E,F,J,K). To determine whether SIRT1 is essential for the SPD‐induced inhibition of mitochondrial fission, we assessed PGAM5 and p‐DRP1(s637)/DRP1 levels after silencing the *SIRT1* gene or EX527 pretreatment, finding that PGAM5 increased and p‐DRP1(s637)/DRP1 levels decreased, and the results of si‐Control group were opposite (Figure 7A–C,G–I). It is further suggested that PGAM5–DRP1‐mediated mitochondrial fission is a key pathological feature of CIRI. SPD, as a SIRT1 activator, alleviated mitochondrial fission and exerted antinecroptotic effects through the SIRT1–RIP1/PGAM5–DRP1 pathway.

Unlike p‐DRP1(s637), p‐DRP1(s616) promoted mitochondrial fission and fragmentation via phosphorylation, and the p‐DRP1(s616)/DRP1 ratio was significantly increased in OGD/R cells. While SPD treatment slightly reduced the levels of p‐DRP1(s616)/DRP1 in vitro, silencing *SIRT1* counteracted this effect (Figure 7D).

## 3 Discussion

In this study, we established MCAO/R and OGD/R models in vivo and in vitro, respectively, to investigate the neuroprotective effects of SIRT1 in CIRI and the underlying mechanisms. First, we demonstrated that the reduction in SIRT1 expression, along with its weakened interaction with RIP1, facilitated necrosome formation and upregulated the expression of RIP1, RIP3, and p‐MLKL/MLKL, thereby exacerbating necroptosis. Second, the downstream signaling molecule PGAM5 was activated, leading to the dephosphorylation of DRP1 at Ser637, which promoted mitochondrial fission and dysfunction, further aggravating CIRI. Moreover, SPD, a potent SIRT1 activator, exhibited significant neuroprotective effects by inhibiting both mitochondrial fission and necroptosis in vivo and in vitro, effectively ameliorating neurological deficits. In summary, our study provides novel insights into the role of SIRT1 in alleviating mitochondrial fission and necroptosis during CIRI via the SIRT1–RIP1 pathway. Additionally, we identified that targeting SIRT1 with TCM, such as SPD, held promise as a potential therapeutic strategy for ischemic stroke.

The pathophysiological processes underlying CIRI are complex, with extensive research identifying necroptosis, mediated by the RIP1–RIP3–p‐MLKL pathway, as a central mechanism [26, 27]. CIRI can trigger TNFα‐mediated necroptosis, in which RIP1 dissociates from its cytoplasmic complex, leading to its activation. Activated RIP1 interacts with RIP3 to form necrosomes, which then activate RIP3, resulting in MLKL phosphorylation. Phosphorylated MLKL forms oligomers that translocate to the cell membrane in association with PGAM5, ultimately causing membrane damage. P‐MLKL serves as the key mediator of necroptosis, promoting the influx of ions such as Na^+^ and Ca^2+^, leading to cell swelling and rupture [9, 26]. Thus, targeting necroptosis has emerged as a promising therapeutic approach for CIRI [12]. In this study, we observed a decrease in SIRT1 expression and an increase in RIP1, RIP3, and p‐MLKL/MLKL levels in rats subjected to MCAO/R and in HT‐22 cells exposed to OGD/R. Notably, these changes were reversed by SPD treatment.

SIRT1, known for modulating various forms of programmed cell death during the acute phase [28, 29, 30]. and for promoting angiogenesis and neurogenesis in later stages [31], has emerged as a promising therapeutic target for ischemic stroke. In our previous studies [23, 24, 25], SPD, a TCM prescription, was shown to act as a potent SIRT1 activator, upregulating its expression, which aligns with the findings of this study. Recent insights underscore the multifaceted role of SIRT1 in cell survival. For instance, propofol has been reported to reduce RIP1, RIP3, and MLKL protein levels by enhancing SIRT1 expression, thereby alleviating necroptosis in renal ischemia–reperfusion injury [32]. In Kupffer cells, the AMPK–SIRT1 pathway activates TTP, which suppresses TNFα production and subsequently decreases RIP1 expression, mitigating necroptosis [18]. However, in contrast to SIRT1's protective role in other diseases, increased SIRT1 expression has been shown to be associated with enhanced necroptosis in the MCAO/R rat model [19]. Given this contradictory evidence, unraveling the precise role of SIRT1 in necroptosis during CIRI remains an intriguing challenge, worthy of further investigation. Our findings confirmed the beneficial effects of increased SIRT1 levels in mitigating necroptosis, as demonstrated by interventions using the SIRT1 inhibitor EX527 and siRNA–SIRT1. Necroptosis induced by TNFα during CIRI triggers the release of proinflammatory factors, some of which are only weakly and temporarily responsive to TNFα. In contrast, necroptotic signaling and RIP1 activation drive more sustained and robust inflammatory responses [33]. As a key regulator, RIP1 orchestrates signaling cascades triggered by toll‐like receptor 4 (TLR4), TNFR1, and TLR3, influencing the NF‐κB and MAPK pathways and determining cell fate by directing apoptosis or necroptosis [34]. Co‐IP assays revealed an interaction between SIRT1 and RIP1, which was diminished in the MCAO/R and OGD/R models, but enhanced following SPD treatment. IF analysis further confirmed the colocalization of RIP1 and SIRT1 in the cytoplasm. To the best of our knowledge, this is the first study to specifically highlight the role of the SIRT1–RIP1 interaction in necroptosis during CIRI, both in vitro and in vivo.

The precise target site of SIRT1 action is critical for modulating the activity of its target proteins. While this study has demonstrated that the SIRT1–RIP1 pathway may be an effective regulator of necroptosis, the mechanisms for enhancing the interaction between these two proteins remain unclear. A previous study identified two deacetylation sites on RIP1, manipulated by SIRT1, and proposed the existence of a RIP1–SIRT1–histone acetyltransferase 1 pathway. The death domain of RIP1 contains lysine residues (K596–K599) that are more acetylated when SIRT1 is inhibited, thereby altering RIP1‐mediated cell death. However, the relationship between phosphorylation and acetylation in RIP1‐mediated cell death remains uncertain [20]. We speculate that there may be a dynamic balance between the phosphorylation and acetylation sites of RIP1. It is possible that deacetylation serves as a catalyst by creating conditions that enable phosphorylation, which in turn activates necroptosis signaling. Through this mechanism, the SIRT1–RIP1 pathway may inhibit necrosome formation by limiting RIP1 activity, allowing cells to respond to death signals with precision via this intricate regulatory machinery. Interestingly, recent studies have identified an alternative necroptotic pathway involving RIP1, which is strongly driven by ROS and independent of TNFα signaling. In this pathway, ROS accumulation triggers RIP1 autophosphorylation and the recruitment of RIP3 into necrosomes, facilitating necroptotic cell death [35, 36]. It is plausible that ROS acts as a mediator between SIRT1 and RIP1. However, SIRT1 activation has also been shown to promote the deacetylation of autophagy‐related gene 5 (Atg5), enhancing RIP1–RIP3 interactions and exacerbating necroptosis in hepatic stellate cells [37]. This finding highlights the dual role of autophagy in both cell survival and death. In conclusion, the SIRT1–RIP1 pathway plays a pivotal role in regulating necroptosis, but further investigation is needed to unravel the complex mechanisms involved.

Mitochondria, as dynamic organelles, undergo structural damage and functional impairment in response to pathological conditions such as ischemia–hypoxia, stress, and reperfusion [38]. Therefore, maintaining mitochondrial integrity is crucial in addressing CIRI. DRP1, a key GTPase involved in mitochondrial fission, translocates from the cytoplasm to the outer mitochondrial membrane and oligomers [39] in response to mitochondrial stress [40]. Dephosphorylation of DRP1 at Ser636/637 triggers a conformational change in DRP1 oligomers, leading to the formation of constriction loops around mitochondria [41]. Excessive mitochondrial fission not only causes morphological changes but also disrupts the mitochondrial membrane, ultimately leading to cell death. Our findings indicated that increasing SIRT1 levels inhibited mitochondrial fission and dysfunction, suggesting a potential restoration of mitochondrial dynamics. Previously, SIRT1 was primarily recognized for enhancing mitochondrial function by activating peroxisome proliferator‐activated receptor γ coactivator‐1α (PGC‐1α), a key regulator of mitochondrial biogenesis, particularly in the brain [42]. However, less attention has been given to other roles of SIRT1 in mitochondrial quality control. Recent studies have highlighted SIRT1's involvement in regulating mitochondrial autophagy [43, 44]. and mitochondrial fission [45, 46]. Inhibition of SIRT1 promotes the acetylation of mitochondrial proteins, which impedes the recruitment of ubiquitin and LC3 for mitophagic degradation [43]. Mitochondrial fission can be suppressed and fusion promoted via the AMPK–SIRT1–Forkhead box protein O1 pathway [45]. Additionally, when SIRT1 translocates from the nucleus to the mitochondria, it deacetylates and activates SIRT3, preserving mitochondrial structure and function, and ultimately mitigating neural injury caused by reperfusion [46]. Our further exploration was undertaken to uncover the mechanism underlying how SIRT1 alleviates mitochondrial fission in CIRI.

Excessive mitochondrial fission is an early and necessary step in necroptosis. Research into the relationship between mitochondrial fission and necroptosis was inspired by the detection of RIP1, RIP3, and p‐MLKL in mitochondrial fractions [47]. Previous work by Cao et al. [48] suggested that RIP1 may promote DRP1‐mediated mitochondrial fission during skeletal muscle I/R injury. More recent studies have supported these findings [49, 50]. PGAM5, a downstream regulator of the RIP1–RIP3–p‐MLKL necrosomes, enhances mitochondrial fission by dephosphorylating p‐DRP1 at Ser637 [51] and Ser636 [11]. The fragmentation and dysfunction of mitochondria lead to a rapid increase in ROS and the escape of mitochondrial deoxyribonucleic acid (mtDNA) [52]. ROS has recently been shown to reciprocally boost the autophosphorylation of RIP1 [35, 36], exacerbating the plasma membrane rupture, pore formation, and the release of DAMPs induced by MLKL, such as mtDNA, adenosine triphosphate (ATP), interleukin 33 (IL‐33), and IL‐1α [10]. And then these reactions recruit immune‐inflammatory cells to the damaged tissues and initiate NF‐kB signaling transduction under the highly regulated control of RIP1, activating inflammatory responses that exacerbate diseases [53]. Therefore, healthy mitochondria can resist necroptosis. PGAM5 knockdown has been shown to inhibit mitochondrial fission and necroptosis, restoring MMP and ATP production in damaged primary cortical neurons [54]. Our study, which involved Western blot and Co‐IP analyses, revealed that PGAM5 levels were increased and p‐DRP1(s637)/DRP1 expressions were reduced in both in vitro and in vivo CIRI models. These findings suggest that PGAM5 interacts with DRP1, dephosphorylating DRP1(s637) and contributing to mitochondrial dysfunction in CIRI. Importantly, elevated SIRT1 levels and their interaction with RIP1 inhibited necroptosis signaling, thereby preventing the interaction between PGAM5 and DRP1. SPD treatment promoted the restoration of mitochondrial characteristics, such as perimeter, roundness, and aspect ratio, indicating a reduction in pathological mitochondrial fission and fragmentation. Furthermore, SIRT1 activation increased MMP levels and reduced ROS accumulation, demonstrating the restoration of mitochondrial function. However, these beneficial effects were diminished by SIRT1 suppression using EX527 and siRNA–SIRT1, suggesting that SIRT1 alleviates mitochondrial fission and exerts antinecroptosis effects in CIRI via the SIRT1–RIP1/PGAM5–DRP1 pathway. Interestingly, PGAM5 may also regulate mitochondrial dynamics independently of DRP1 activity, indicating the existence of alternative pathways. For example, PGAM5 has been shown to promote mitochondrial network formation under normal conditions by acting as a phosphatase for MFN2, a key regulator of mitochondrial fusion. In response to mitochondrial stress, the PGAM5–MFN2 interaction weakens, leading to PGAM5 translocation to the cytoplasmic lysosome, where it binds to and dephosphorylates DRP1 [55].

In contrast to SIRT1, which is distributed in both the cytoplasm and nucleus, SIRT6 is predominantly localized in the nucleus and is involved in DNA repair, telomere maintenance, and other cellular processes [56]. Interestingly, SIRT6 has also been detected on the endoplasmic reticulum (ER) under stressful conditions, suggesting that its role extends beyond the nucleus [57]. Mechanistically, overexpression of SIRT6 has been shown to promote ERK1/2‐driven phosphorylation of DRP1 at Ser616, a key step in inducing mitochondrial fission [58]. However, it remains unclear whether SIRT1 influences DRP1(s616). To investigate this, we conducted a preliminary in vitro assessment of DRP1(s616). Notably, SIRT1 upregulation led to a modest reduction in p‐DRP1(s616)/DRP1 levels, an effect that was abolished when the SIRT1 gene was silenced. Other studies have shown that melatonin inhibits mitochondrial fission by downregulating DRP1 expression through the SIRT1–PGC1α pathway [59], while also promoting MFN2 transcription to enhance mitochondrial fusion [60]. Collectively, our preliminary findings suggest that SIRT1 may exert a regulatory role in modulating DRP1(s616)‐mediated mitochondrial fission.

Importantly, since DRP1(s616) phosphorylation occurs independently of PGAM5, this raises a critical question: Can necroptosis only be initiated by PGAM5‐dependent mitochondrial fission, or can it also be triggered by PGAM5‐independent mechanisms? [61] While mitochondria are known to play a role in necroptosis, there is ongoing debate about their specific involvement in this form of cell death. Some studies suggest that mitochondria are not essential for necroptosis, and the regulatory role of PGAM5 in necroptosis may vary depending on the cellular context and specific disease conditions. For instance, dephosphorylated DRP1 has been shown to translocate to mitochondria in response to high glucose levels, where it functions as a fission regulator and drives necroptosis [36]. In renal [50] and testicular injury models [62], PGAM5–DRP1 signaling induces mitochondrial dysfunction and amplifies necroptosis, exacerbating tissue damage. Conversely, the absence of PGAM5 has been linked to increased cellular senescence. The interferon‐β (IFN‐β)–PGAM5–DRP1(s622) pathway strengthens the mitochondrial–ER platform, promoting the efficient sequestration and degradation of old or damaged mitochondria [63]. Deletion of PGAM5 leads to the accumulation of phosphorylated, inactive DRP1, impairing mitochondrial fission and turnover. This contributes to the decline in antioxidant capacity associated with aging, partially driven by the activation of IFN regulatory factor/IFN‐β signaling [64]. Furthermore, in cardiac ischemia–reperfusion injury, elevated levels of PGAM5 have been associated with increased mitochondrial autophagy, which reduces necroptosis in cardiomyocytes [65].

In the present study, although it has been established that the interaction between SIRT1 and RIP1 is negatively correlated with the formation of the necrosomes, the precise mechanism underlying the connection between SIRT1–RIP1 phosphorylation and acetylation remains unknown. From the molecular level, this makes it difficult for us to accurately describe how these two modifications are precisely regulated in the SIRT1–RIP1 pathway. For example, we are still unclear about which upstream signaling molecules or enzymes are directly involved in the conversion of RIP1 phosphorylation and acetylation states, as well as their action sequence and conditions. Currently, the methods we have used, such as Co‐IP and IF analysis, do provide qualitative and quantitative insights into the SIRT1–RIP1 interaction. However, these methods have certain limitations and may be unable to detect low abundance proteins or transient modification changes. Furthermore, SIRT1 can alter gene transcription levels via deacetylating transcription factors, but this crucial function was not investigated in our study. In future studies, we intend to integrate multiple approaches to more comprehensively evaluate SIRT1‐regulated necroptotic signals. For example, bioinformatics analysis was performed to identify potential genes involved in necrotic apoptosis and regulated by SIRT1 transcription. Mass spectrometry‐based quantitative proteomics technology, for example, may detect transitory protein modification changes with high accuracy. Meanwhile, by integrating live cell imaging with fluorescence resonance energy transfer technology, the real‐time dynamic localization of SIRT1–RIP1 in cells can be detected, revealing their real‐time behaviors in different cell states.

## 4 Conclusion

**FIGURE 8:**
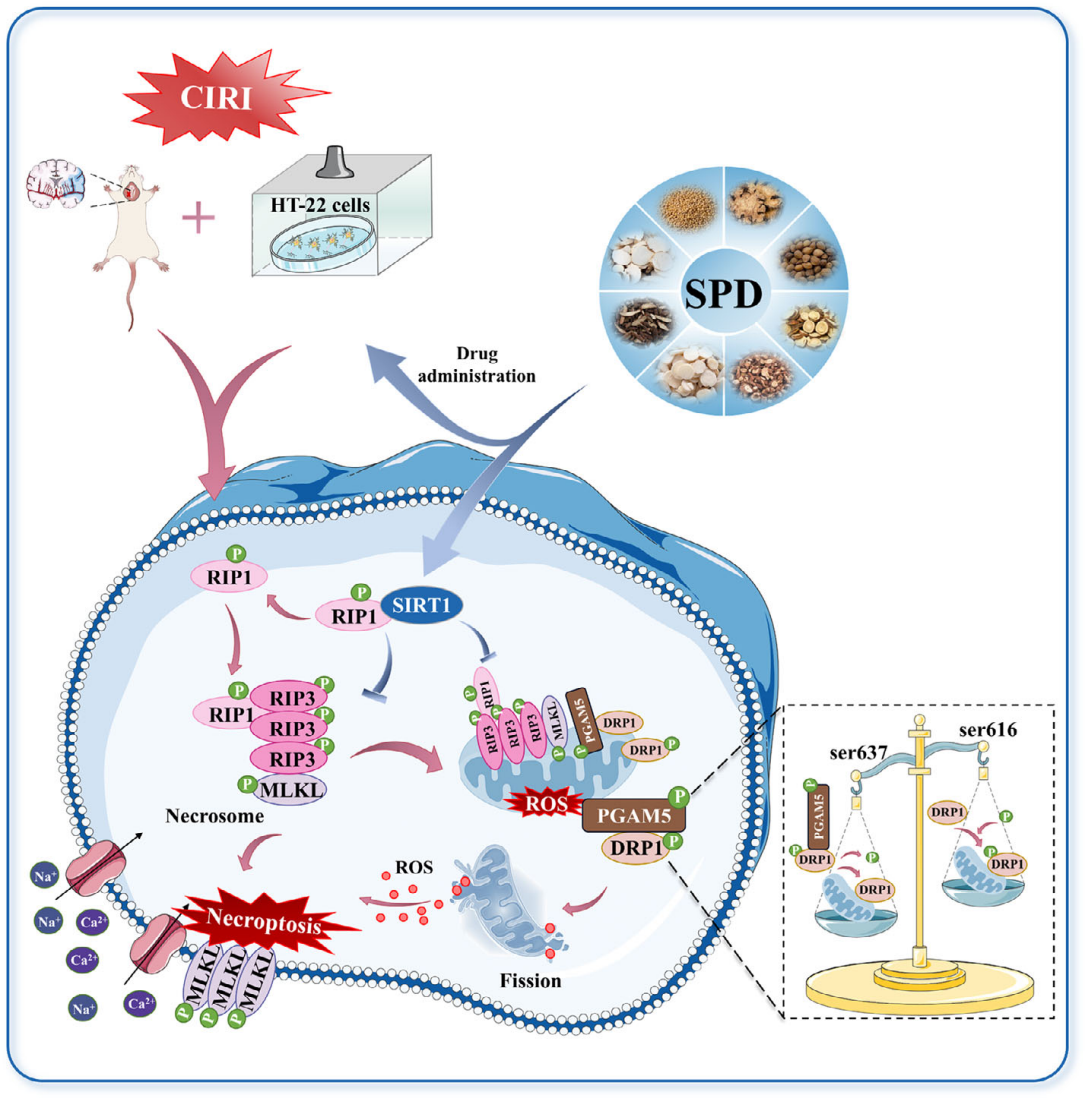
SIRT1 restores mitochondrial function and exerts antinecroptosis role via the SIRT1–RIP1 signal.

In summary, our study demonstrates that the weakened interaction between SIRT1 and RIP1 promotes necrosome formation, exacerbating necroptosis. Additionally, activation of the PGAM5–DRP1 pathway intensifies mitochondrial fission and dysfunction, further aggravating CIRI. However, SPD, a traditional herbal formula, serves as a potent SIRT1 activator, reversing these pathological changes (Figure 8). Elucidating the precise role of the SIRT1–RIP1 interaction in necroptosis will not only enhance our understanding of the pathological mechanisms underlying CIRI but also pave the way for developing novel therapeutic strategies using TCM that targets SIRT1 for the treatment of CIRI.

## 5 Materials and Methods

### 5.1 Animals in Vivo

This study was conducted in accordance with the National Institutes of Health Guide for the Care and Use of Laboratory Animals. All experimental procedures were supervised by the Animal Experiment Center of Hunan University of Chinese Medicine and approved by the Ethics Committee of the Animal Laboratory of Hunan University of Chinese Medicine (Permit Number: LL2022062203).

Male Sprague–Dawley rats (260–280 g) were obtained from the Laboratory Animal Center of Hunan University of Traditional Chinese Medicine (Hunan, China). The rats were housed in a controlled environment (a 12‐h light–dark cycle, 50% humidity, and a temperature of 21–25°C), with access to food and water.

### 5.2 MCAO/R Model and Drug Administration in Vivo

The right MCAO/R model was established following the procedure described by Longa et al. [66]. After a week of acclimatization, the rats were fasted for 12 h prior to surgery and anesthetized with 2% pentobarbital sodium (50 mg/kg). The rats were positioned supine on a surgical plate, and a midline ventral neck incision was made. The right common carotid artery and external carotid artery were dissected and ligated. A nylon thread was inserted into the internal carotid artery to block the origin of the middle cerebral artery, inducing MCAO. After 1.5 h of transient focal cerebral ischemia, the nylon thread was withdrawn to restore cerebral blood flow.

Following acclimatization, the rats were randomized into sham group, I/R group, I/R+SPD (1.35 g/kg) group, I/R+SPD (2.7 g/kg) group, and I/R+SPD (2.7 g/kg) +EX527 group. Rats in the SPD groups received SPD concentrate via gavage, while the remaining groups received equivalent amounts of saline by gavage once daily for 7 days prior to MCAO/R. CIRI was induced in all groups except the sham group through MCAO/R surgery.

### 5.3 Cerebral Blood Flow Measurements in Vivo

To assess cerebral ischemia and reperfusion, rCBF was measured at three‐time points: before ischemia, immediately after ischemia, and 24 h after reperfusion. Briefly, the rat's skull was carefully exposed and gently abraded until the blood vessels were clearly visible. rCBF was monitored using a MoorFLPI Full‐field Laser Perfusion Imager. Baseline rCBF values were recorded 5 min prior to the onset of ischemia.

### 5.4 Evaluation of Neurological Deficits in Vivo

Neurological deficits were evaluated 24 h after reperfusion using the Zea Longa scoring system [67]. A blind investigator assigned scores based on the severity of neurological impairment, with higher scores reflecting more significant deficits.

### 5.5 Morris Water Maze Test in Vivo

Learning and memory abilities were assessed using the Morris water maze. Rats underwent training in the maze for 1 week prior to ischemia modeling. Each day, rats were placed in the pool and tasked with locating a hidden platform in the target quadrant, with a clockwise sequence followed. The time taken to find the platform within 1 min was recorded as the escape latency. After the training phase, the platform was removed, and rats were placed in the pool starting from the opposite quadrant. During the 60‐s test period, escape latency, the number of times the rats crossed the previous platform location, and the time and distance spent in the target quadrant were recorded.

### 5.6 Measurement of Infarct Volume in Vivo

To determine the infarct volume, rats were euthanized following 1.5 h of ischemia and 24 h of reperfusion. Coronal brain sections were cut at 2‐mm intervals and stained with 2% TTC for 30 min at 37°C. After staining, the sections were fixed in 4% paraformaldehyde and photographed. Cerebral infarct areas were quantified using Image‐Pro Plus 6.0 by an examiner blinded to the experimental groups.

### 5.7 HE Staining and Nissl Staining in Vivo

Histological evaluation of the cortical and hippocampal CA1 regions was performed using HE and Nissl staining. Twenty‐four hours postreperfusion, brains from all five groups were collected and immediately fixed in paraffin. Coronal sections (5 µm thick) were prepared and stained with HE and Nissl to visualize the morphology of the cortex and hippocampal CA1 region. The stained sections were examined using a Nikon light microscope (Japan) for analysis.

### 5.8 TEM Observation of in Vivo Findings

The ultrastructure of mitochondria was examined using TEM. The cortical tissue was carefully extracted to minimize mechanical damage. Small pieces of cortical tissue (1 mm^3^) were rapidly sectioned with a sharp blade (within 1 min) and immediately immersed in 2.5% glutaraldehyde for fixation. The specimens were stored at 4°C for 2–4 h. After fixation, the samples were embedded in resin blocks, from which ultrathin sections (60–80 nm) were obtained using an ultramicrotome. The sections were double stained with uranyl acetate and lead citrate, then observed under a TEM, and images were captured for analysis. Mitochondrial morphology was assessed in 3–6 fields of view using Image J Pro Plus. Parameters such as mitochondrial perimeter, roundness, and aspect ratio were measured to quantify morphological changes. Roundness was calculated by squaring the mitochondrial perimeter and dividing it by 4*π*, while the aspect ratio was determined as the ratio of the major to the minor axes of each mitochondrion.

### 5.9 RT‐qPCR in Vivo

RT‐qPCR was implemented to analyze the total ribonucleic acid (RNA) extracted from cerebral cortex samples. Reverse transcription was carried out utilizing kit No. G3337 to synthesize complementary DNA from the total RNA. The specific primer sequences utilized for RT‐qPCR are listed in Table S3 (Wuhan Saiville Biotechnology Co., Ltd). The polymerase chain reaction conditions were set as follows: an initial denaturation at 95°C for 30 s, followed by 40 cycles of 95°C for 15 s and 60°C for 30 s. A final temperature ramp from 65 to 95°C at a rate of 0.5°C per cycle was applied, during which fluorescence signals were collected. mRNA expression levels were quantified employing the 2^−ΔΔCT^ method.

### 5.10 Cell Culture in Vitro

Mouse hippocampal HT‐22 cells were cultured in Dulbecco's modified Eagle's medium (DMEM; PM150210; Pricella, China) enriched with 10% fetal bovine serum (FBS; P30‐3306; PAN, USA). The cells were maintained in an incubator at 37°C with 5% CO_2_, and the growth medium was refreshed every 12 h.

### 5.11 OGD/R Model, Transfection, and Drug Administration in Vitro

I/R injury was modeled in vitro using OGD/R. HT‐22 cells were cultured in glucose‐free DMEM (PM150270; Pricella, China) and subjected to hypoxic conditions (5% CO_2_, 94% N_2_, and 1% O_2_) for 6 h. Following this, the cells were returned to normal DMEM and incubated for 24 h to simulate reperfusion. For transfection, Lipofectamine 2000 Transfection Reagent (11668019; Invitrogen, USA) was applied. The sequences of the siRNA applied were as follows:

si‐SIRT1‐1 sense (UUUUCCUUCCUUAUCUGACAA).

si‐SIRT1‐2 sense (AUUUUCUCACUGUUUCUGCAA).

si‐SIRT1‐3 sense (AAAUCUUUAAGAAUUGUUCGA).

After 24 h of siRNA transfection, SIRT1 protein expression was evaluated by western blot analysis and quantified using Image J software. The cell experiments were divided into: control group, OGD/R group, OGD/R+SPD group, OGD/R+SPD+si‐SIRT1 group, and OGD/R+SPD+si‐Control group.

### 5.12 Annexin V and PI Staining in Vitro

The rates of necroptosis and apoptosis were measured by flow cytometry after staining the cells with the Annexin V‐FITC/PI Apoptosis Detection Kit (A211‐01; Vazyme, China). In this assay, Annexin V‐positive cells were identified as apoptotic, PI‐positive cells were classified as necrotic, and cells negative for both Annexin V and PI were considered normal and unaffected.

### 5.13 Detection of ROS Accumulation and MMP in Vitro

To assess intracellular ROS levels, HT‐22 cells were stained with H2DCFDA (Ros100; ZETA) in the dark, and images were captured using confocal laser scanning microscope (SP8; LEICA, China). For the measurement of MMP, the JC‐1 MMP assay kit (M8650; Solarbio, China) was employed. After the designated treatments, the cells were washed twice with FBS and incubated with JC‐1 solution for 20 min at 37°C. MMP was quantified by calculating the ratio of red fluorescence (indicative of high MMP) to green fluorescence (indicative of low MMP) using flow cytometry.

### 5.14 Assessment of Mitochondrial Morphology in Vitro

Mitochondrial morphology in HT‐22 cells was evaluated using the MitoTracker Red CMXRos probe (100 nmol/L, 30 min at 37°C). Images were acquired using a confocal laser scanning microscope (SP8; LEICA). Mitochondrial quantity was analyzed, and the percentage of cells displaying fragmented mitochondria (small and round) was determined.

### 5.15 IF in Vitro

Cells were fixed and permeabilized using 0.13% Triton X‐100 in PBS for 5 min. Following permeabilization, sections were blocked with 5 mg/mL bovine serum albumin for 1 h. Cells were then incubated with primary antibodies targeting SIRT1 and RIP1 at 37°C for 2 h. After PBS rinsing, the cells were incubated with secondary antibodies in the dark for 1 h at room temperature. Nuclei were stained with 4′,6‐diamidino‐2‐phenylindole before imaging with a confocal microscope (SP8; LEICA, Germany).

### 5.16 Western Blot in Vivo and in Vitro

Total proteins were extracted from cerebral cortex tissues or cultured cells following the manufacturer's instructions. Equal amounts of protein were separated by sodium dodecyl sulfate‐polyacrylamide gel electrophoresis (SDS‐PAGE) and subsequently transferred onto 0.45 µm PVDF membranes. The membranes were blocked with 5% nonfat milk at room temperature for 30 min and then incubated with the appropriate primary antibodies overnight at 4°C. After washing, membranes were incubated with horseradish peroxidase‐conjugated goat anti‐rabbit secondary antibodies for 30 min at room temperature, followed by additional washing with TBST. The following primary antibodies were utilized for in vivo experiments: SIRT1 (1:3000, cat: ab189494; Abcam), RIP1 (1:3000, cat: 3493; CST), RIP3 (1:3000, cat: 15828; CST), phospho‐MLKL (S345) (1:1000, cat: AP1244; Abclonal), MLKL (1:3000, cat: A19685; Abclonal), PGAM5 (1:3000, cat: 63454; CST), phospho‐DRP1 (S637) (1:1000, cat: 4867; CST), DRP1 (1:3000, cat: 12957‐1‐AP; Proteintech), and β‐actin (1:3000, cat: GB15001; Servicebio). For in vitro experiments, the primary antibodies utilized were: SIRT1 (1:1000, cat: 8469; CST), RIP1 (1:1000, cat: 17519‐1‐AP; Proteintech), RIP3 (1:2000, cat: 17563‐1‐AP; Proteintech), phospho‐MLKL (S345) (1:800, cat: ab196436; Abcam), MLKL (1:3000, cat: 66675‐1‐Ig; Proteintech), PGAM5 (1:2000, cat: 68116‐1‐Ig; Proteintech), phospho‐DRP1 (S616) (1:800, cat: 4494; CST), phospho‐DRP1 (S637) (1:1000, cat: 4867; CST), DRP1 (1:2000, cat: 12957‐1‐AP; Proteintech), and β‐actin (1:5000, cat: 20536‐1‐AP; Proteintech). Protein expression levels were analyzed using image analysis software.

### 5.17 Co‐IP in Vivo and in Vitro

Co‐IP was adopted to examine protein‐protein interactions. Antibodies were first coupled to Protein G magnetic beads and incubated at 37°C for 1 h, followed by a washing step to remove excess reagents. The magnetic bead‐antibody complex was then added to each sample for incubation overnight at 4°C, allowing the antibody to bind its target proteins. After incubation, the magnetic beads were separated and washed several times to remove unbound components. The samples were then mixed with 100 µL of SDS‐PAGE loading buffer and heated to 95°C for 5 min. The supernatant was collected for further analysis by Western blot.

### 5.18 Statistical Analysis

All experimental data were analyzed utilizing SPSS 26.0 statistical software (IBM Corp., Armonk, NY, USA), and graphs were generated with GraphPad Prism 9.5 (GraphPad Software). Data were expressed as the mean ± standard error of the mean. For multigroup comparisons, one‐way analysis of variance was utilized. A *p* value of <0.05 was deemed statistically significant.

## Author Contributions

W.L.Z. and Z.G.M. conceptualized and designed the study. X.W. performed the investigation, visualization, and formal analysis, and drafted the manuscript. H.J.G. carried out the investigation, visualization, and formal analysis, and added it to the manuscript. G.S.H., H.Y.L., and L.P.G. were responsible for partial investigation. J.Y.L. and P.M. revised the manuscript. All authors have read and approved the final manuscript.

## Ethics Statement

The experimental protocol was established according to the ethical guidelines of the National Institute of Health Guide for the Care and Use of Laboratory Animals and was approved by the Ethics Committee of the Animal Laboratory of Hunan University of Chinese Medicine (Permit Number: LL2022062203).

## Conflicts of Interest

The authors declare no conflicts of interest.

## Supporting information



Supporting Information

## Data Availability

With the consent of the corresponding author, the data in this article are shared.
