# Supplementary material for: SIRT1 Alleviates Mitochondrial Fission and Necroptosis in Cerebral Ischemia/Reperfusion Injury via SIRT1–RIP1 Signaling Pathway
Source: MedComm (2020). 2025 Feb 24;6(3):e70118. doi: 10.1002/mco2.70118 (PMC11850763; doi:10.1002/mco2.70118)
Supplement: Supplementary file 1 — Supporting Information [file MCO2-6-e70118-s001.docx]

**Supplementary Information**

**SIRT1 alleviates mitochondrial fission and necroptosis in** **cerebral ischemia/reperfusion injury *via* SIRT1-RIP1 signaling pathway**

Xuan Wei^1, #^, Hanjing Guo^1, #^, Guangshan Huang^1^, Haoyue Luo^1^, Lipeng Gong^1^, Pan Meng^1^, Jiyong Liu^2^, Wenli Zhang^3, *^, Zhigang Mei^1, 4, *^

1 Key Laboratory of Hunan Province for Integrated Traditional Chinese and Western Medicine on Prevention and Treatment of Cardio-Cerebral Diseases, College of Integrated Traditional Chinese and Western Medicine, Hunan University of Chinese Medicine, Changsha 410208, Hunan, China.

2 Hunan Provincial Key Laboratory of Traditional Chinese Medicine Diagnostics, Hunan University of Chinese Medicine, Changsha 410208, Hunan, China.

3 School of Pharmacy, Hunan University of Chinese Medicine, Changsha 410208, Hunan, China.

4 Third-Grade Pharmacological Laboratory on Chinese Medicine Approved by State Administration of Traditional Chinese Medicine, College of Medicine and Health Sciences, China Three Gorges University, Yichang 443002, Hubei, China.

^#^ These authors contributed equally to this work.

^*^ Corresponding authors

Zhigang Mei, College of Integrated Traditional Chinese and Western Medicine, Hunan University of Chinese Medicine, No.300, Xueshi Road, Changsha 410208, Hunan, China.

Wenli Zhang, School of Pharmacy, Hunan University of Chinese Medicine, No.300, Xueshi Road, Changsha 410208, Hunan, China.

E-mail:

meizhigang@hnucm.edu.cn (Zhigang Mei);

zhangwenli@hnucm.edu.cn (Wenli Zhang).

**Materials and Methods**

**Cell culture and transfection *in vitro***

Mouse hippocampal HT-22 cells were cultured in Dulbecco’s modified Eagle’s medium (DMEM, PM150210, Pricella, China) enriched with 10% fetal bovine serum (FBS, P30-3306, PAN, USA). The cells were maintained in an incubator at 37°C with 5% CO_2_, and the growth medium was refreshed every 12 hours. For transfection, Lipofectamine 2000 Transfection Reagent (11668019, Invitrogen, USA) was applied.

**qPCR detection of si-SIRT1 interference efficiency *in vitro***

qPCR was implemented to analyze the total RNA extracted from HT-22 samples. The specific primer sequences utilized for qPCR are listed in Table S1 (Wuhan Saiville Biotechnology Co., Ltd). The PCR conditions were set as follows: an initial denaturation at 95°C for 30 seconds, followed by 40 cycles of 95°C for 15 seconds and 60°C for 30 seconds. A final temperature ramp from 65°C to 95°C at a rate of 0.5°C per cycle was applied, during which fluorescence signals were collected. mRNA expression levels were quantified employing the 2^-ΔΔCT^ method.

**Results**


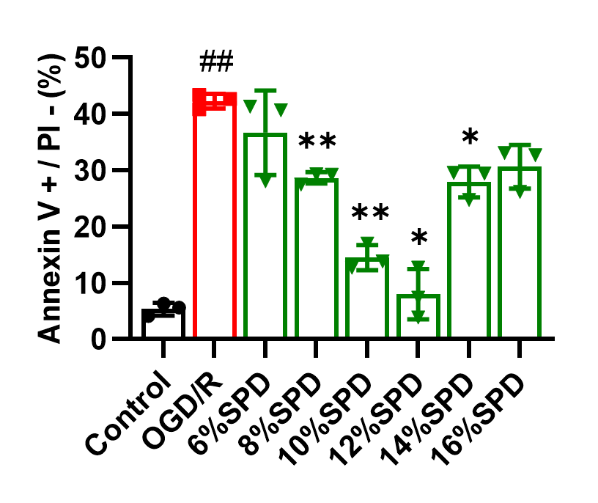


**Figure S1.** Annexin V and PI staining under different doses of SPD. Annexin V + cells represented apoptosis (n=3). The results are expressed as the mean ± SEM. ^##^*p* < 0.01; Control vs OGD/R. ^*^*p* < 0.05, ^**^*p* < 0.01; OGD/R vs OGD/R+SPD.


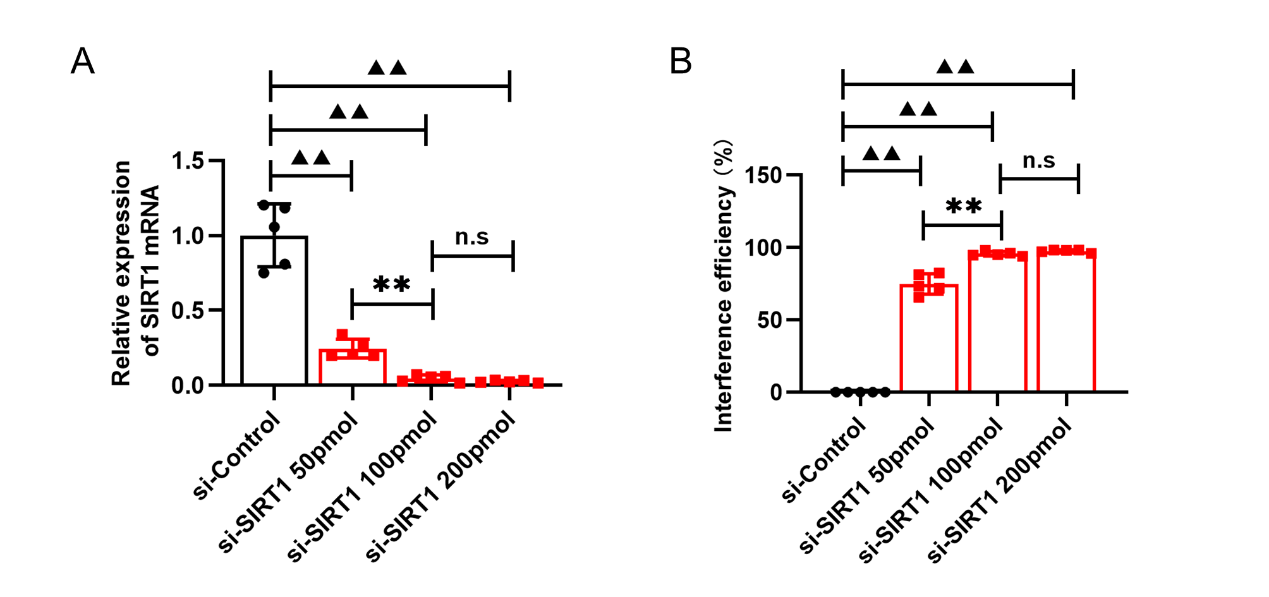


**Figure S2.** qPCR detection of si-SIRT1 interference efficiency. (A) qPCR was undertaken to detect the interference efficiency of si-SIRT1 (Table S1). The SIRT1 mRNA expression levels in the 50pmol, 100pmol, and 200pmol groups were considerably lower than those of the si-Control group (*p* < 0.01). (B) 50 pmol, 100 pmol, and 200 pmol had interference efficiencies of 74.88±8.48, 95.62±2.03, and 97.45±1.34, in that order. Given that there was no discernible statistical difference between 100 pmol and 200 pmol, the experiment was conducted with 100 pmol (n=5). The results are expressed as the mean ± SEM. ^▲▲^*p* < 0.01; si-Control vs si-SIRT1. ^**^*p* < 0.01; 50pmol vs 100pmol. ^n.s^*p* > 0.05; 100pmol vs 200pmol.


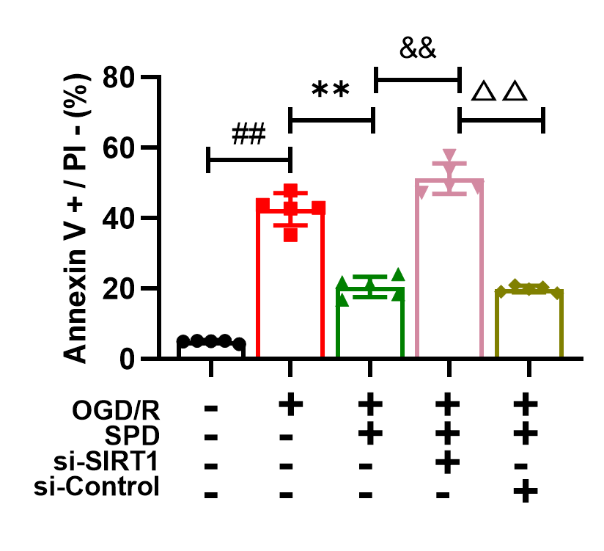


**Figure S3.** Representative images and analysis of Annexin V and PI staining. Annexin V + cells represented apoptosis (n=5). The results are expressed as the mean ± SEM. ^##^*p* < 0.01; Control vs OGD/R. ^**^*p* < 0.01; OGD/R vs OGD/R+SPD. ^&&^*p* < 0.01; OGD/R+SPD vs OGD/R+SPD+si-SIRT1. ^△△^*p* < 0.01; OGD/R+SPD+si-SIRT1 vs OGD/R+SPD+si-Control.

**Table S1. Primer sequence.**

| Primer | 5‘ - 3’ | bp |
| --- | --- | --- |
| Mu-SIRT1-F | TCCTTGGAGACTGCGATGTTA | 154 |
| Mu-SIRT1-R | GGTGGCAACTCTGATAAATGAAC |  |
| Mu-β-actin-F | GAAATCGTGCGTGACATCAAAGA | 185 |
| Mu-β-actin-R | CCCAAGAAGGAAGGCTGGAAAA |  |

**Table S2. qPCR detection of si-SIRT1 interference efficiency.**

| Groups (n=5) | SIRT1 expression | Interference efficiency % |
| --- | --- | --- |
| si-Control | 1.00±0.209 |  |
| si-SIRT1 50pmol | 0.24±0.062 | 74.88±8.48 |
| si-SIRT1 100pmol | 0.04±0.023 | 95.62±2.03 |
| si-SIRT1 200pmol | 0.03±0.008 | 97.45±1.34 |

**Table S3. Primer sequence.**

| **Gene** | **Primer sequences** |
| --- | --- |
| SIRT1 | Forward: 5′-AGATTTCAAGGCTGTTGGTTCC-3′ |
|  | Reverse: 5′-CAGCATCATCTTCCAAGCCATT-3′ |
| RIP1 | Forward: 5′-TCTCCATAGTGCTGAGCCCAAC-3′ |
|  | Reverse: 5′-CCTTGATGCCTGCTTGGGT-3′ |
| RIP3 | Forward: 5′-CCAAATTCCACATACTTTACCCTCC-3′ |
|  | Reverse: 5′-TTCTTCCATCTCCCTGATTCCTT-3′ |
| MLKL | Forward: 5′-TCCCACAAGATTTCCAAGTCAA-3′ |
|  | Reverse: 5′-GCCTCACTATTCCAACACTTTCG-3′ |
| PGAM5 | Forward: 5′-TCCTCTATGACCCGTGCAGT-3′ |
|  | Reverse: 5′-AGGTGGATCCGGTTCAATGG-3′ |
| DRP1 | Forward: 5′-TCCAGCTCATTACCAAGTTTGC-3′ |
|  | Reverse: 5′-CAGATTCTAAGGTTCGCCCAAA-3′ |
| GAPDH | Forward: 5′-CTGGAGAAACCTGCCAAGTATG-3′ |
|  | Reverse: 5′-GGTGGAAGAATGGGAGTTGCT-3′ |
